# Lifetime Stressor Severity and Diurnal Cortisol in Older African American Adults: A Comparison of Three Theoretical Models

**DOI:** 10.1002/dev.70034

**Published:** 2025-03-19

**Authors:** Katherine Knauft, Kristin M. Davis, Yanping Jiang, Grant S. Shields, Nataria Joseph, George M. Slavich, Samuele Zilioli

**Affiliations:** ^1^ Department of Psychology and Neuroscience Baylor University Waco USA; ^2^ Department of Psychology Wayne State University Detroit USA; ^3^ Institute for Health, Health Care Policy and Aging Research, Rutgers The State University of New Jersey New Brunswick USA; ^4^ Department of Family Medicine and Community Health, Rutgers The State University of New Jersey New Brunswick USA; ^5^ Department of Psychological Science University of Arkansas Fayetteville USA; ^6^ Department of Psychology Pepperdine University Malibu USA; ^7^ Department of Psychiatry and Biobehavioral Sciences University of California Los Angeles USA; ^8^ Department of Family Medicine and Public Health Sciences Wayne State University Detroit USA

**Keywords:** African Americans, aging, cortisol, cumulative stress, hypothalamic‐pituitary‐adrenal (HPA) axis, life stress

## Abstract

Multiple theoretical models have been proposed to explain how stressor exposure across the life course relates to the functioning of the hypothalamic‐pituitary‐adrenal (HPA) axis, as indexed by daily cortisol secretion. However, this association remains understudied in African Americans. The present study tested three competing models of stressor severity across the lifespan and diurnal cortisol secretion in a sample of 203 older African American adults. The cumulative model emphasizes total stressor severity across the lifespan, the biological embedding model emphasizes early‐life stressor severity, and the sensitization model instead emphasizes the interaction between early‐life and recent stressor severity. Lifetime stressor severity was assessed using the Stress and Adversity Inventory for Adults (Adult STRAIN). Analyses did not support any of the three models tested but, rather, a stressor characteristics perspective, wherein the severity of exposure to specific stressor characteristics was associated with blunted diurnal cortisol slopes. Sensitivity analyses revealed that early life stressor count, rather than severity, was associated with blunted diurnal cortisol slopes. Rather than supporting one of the three competing models of stressor severity, our findings provide preliminary evidence for a stressor characteristics approach and the biological embedding model when examining how lifetime stressor exposure affects HPA‐axis activity.

## 1 Introduction

Psychological stress is implicated in disease risk (Cohen et al. 1998; Cohen et al. 2007; Gianaros and Wager 2015; Segerstrom and Miller 2004), and dysregulation of the hypothalamic‐pituitary‐adrenal (HPA) axis is a candidate mechanism through which stress may contribute to disease‐related physiological changes (Cohen et al. 2016; McEwen 2017). Cortisol levels are typically high upon waking. Cortisol levels then increase sharply within the first 30 min following awakening, which is known as the cortisol awakening response (CAR). Cortisol levels then decrease throughout the day before reaching their lowest point at bedtime; this decline is referred to as the diurnal cortisol slope (Adam and Kumari 2009). Normative HPA axis function can be altered through repeated activation of the HPA axis in response to stressful circumstances (Lupien et al. 2009; McLaughlin et al. 2015; Stalder et al. 2017). Alterations in HPA axis function, particularly flattening of the diurnal cortisol slope, are associated with poorer physical health (Adam et al. 2017). However, the relative contributions of stressor exposure occurring across the lifespan on HPA axis functioning need to be clarified.

Multiple competing models, including the cumulative model, the biological embedding model, and the sensitization model, have been proposed to account for how stressor exposure across different developmental periods may lead to alterations in daily cortisol secretion (Young et al. 2019). In addition, as individuals may perceive a stressful event in various ways, examining stressor severity provides a unique window into how individuals experience and respond to stressors (Epel et al. 2018). The present study, therefore, tested these three competing models of the relation between stressor severity across the lifespan and diurnal cortisol parameters among older African Americans.

### 1.1 Models of Stressor Severity across the Lifespan and Diurnal Cortisol

The cumulative model emphasizes the total accumulation of stressors across the lifespan as the primary predictor of alterations in health‐relevant physiological processes (Karatsoreos and McEwen 2013; McEwen 1998, 2008). This model posits that the sum of stressor exposure across the lifespan leads to the dysregulation of key physiological systems, including the HPA axis (McEwen 2008). In contrast, the biological embedding model emphasizes early childhood as a period during which health‐relevant physiology is particularly sensitive to perturbation (Doom and Gunnar 2013; Koss et al. 2016; Lupien et al. 2009; McLaughlin et al. 2015). Exposure to stressors during this developmental period is thought to predict dysregulation of the HPA axis in adulthood (Gunnar 2021).

Finally, similarly to the biological embedding model, the sensitization model highlights the developmental importance of childhood for calibrating health‐related biological processes but also emphasizes the role of recent stressors (Daskalakis et al. 2013; Young et al. 2019). This model posits that early stress exposure may increase the vulnerability to poor physiological health following exposure to stress during adulthood. In other words, the sensitization model hypothesizes that the interactive effect of early and recent life stressors best predicts HPA axis dysfunction (Daskalakis et al. 2013).

Young and colleagues (Young et al. 2019) tested these three competing models using the Minnesota Longitudinal Study of Risk and Adaptation (MLSRA; Sroufe et al. 2005), which comprises 19 waves of life stress data collected between birth and age 37 in 90 participants. Race and ethnicity for this sample did not significantly differ from that of the full MLSRA sample, which was 58.4% White (Doom et al. 2023). Daily cortisol data were collected at age 37. The results supported the sensitization model, such that individuals with high early life stressors in combination with high current life stressors had significantly flatter diurnal slopes than the rest of the sample.

### 1.2 Racial Differences in Examinations of Stress and Cortisol

Differences in stressor exposure, health, and associations between stress and health across race have been previously observed. For example, rates of chronic disease and premature mortality are significantly higher among African American adults compared to White Americans (Duru et al. 2012). These documented racial disparities in health outcomes are thought to largely be the result of stress, discrimination, and systemic racism (Sternthal et al. 2011; Van Dyke et al. 2020; Williams 1999), rather than race itself. Indeed, African Americans are more likely to be exposed to a variety of major stressors in financial, residential, health, and relationship domains (Brown et al. 2020). Biological processes linked to stress—such as blood pressure, inflammation, and oxidative stress—also tend to be higher among African Americans (Lewis et al. 2009; Szanton et al. 2012; Tomfohr et al. 2016). For example, African American adolescents, young adults, and older adults consistently have flatter diurnal cortisol slopes than White individuals (Cohen et al. 2006; Deer et al. 2018; DeSantis et al. 2007; Ernst et al. 2021; Fuller‐Rowell et al. 2012; Samuel et al. 2016; Skinner et al. 2011).

Interestingly, prior studies have also found key differences in the associations between stressor exposure and diurnal cortisol slope between African American and White participants, such that stressor exposure was a stronger predictor of diurnal cortisol slopes in White compared to African American participants (Skinner et al. 2011). Therefore, examining the associations between stressors across the lifespan and cortisol is needed to shed light on how major stressors get under the skin within African American adults. However, much of the existing work examining relations between stressors across the lifespan and cortisol has been conducted in predominantly White samples (e.g., Young et al. 2019). As such, it is unclear whether findings from these studies will generalize to African Americans.

### 1.3 Present Study

To address this gap, we tested the cumulative model, biological embedding model, and sensitization model in a sample of African Americans to evaluate the relative contributions of lifetime stressor severity, early life stressor severity, and the interaction between early and recent life stressor severity to diurnal cortisol patterns. Study hypotheses and analyses were preregistered on the Open Science Framework (https://osf.io/asfdv). In brief, based on Young and colleagues’ findings (Young et al. 2019), we hypothesized that the interaction between high early and high recent life stress severity would be associated with a blunted diurnal cortisol slope. As recommended by Adam and Kumari (2009), the associations between lifetime, early life, and recent stressor severity with cortisol at awakening and CAR were also simultaneously examined. Though our hypotheses are specific to the diurnal cortisol slope, this approach provides a more comprehensive assessment of the associations between our predictors and overall diurnal cortisol patterns.

## 2 Method

### 2.1 Participants

Data were derived from the Health among Older Adults Living in Detroit (HOLD) study, which examined links between psychosocial and biobehavioral factors and physical health in a sample of older African American adults living in Detroit, MI (*N* = 211). One person withdrew from the study, and seven participants did not provide daily cortisol samples. Participants who did not have cortisol were excluded from the present analyses, leaving a final analytic sample of 203 participants (*M*_age_ = 67.5 years, SD = 8.4, range = 50–89; 72.4% Female). Participants were recruited between November 2017 and March 2020. Most participants (62%) were recruited through the Institute of Gerontology's Healthy Black Elders Center Participant Research Pool (Mitchell et al. 2020). Additional participants were recruited through snowball sampling and flyers placed in the local community.

### 2.2 Procedure

The study consisted of two home visits, separated by an at‐home period. At the first home visit, participants provided written informed consent and completed questionnaire measures, including the Stress and Adversity Inventory for Adults (Adult STRAIN; Slavich and Shields 2018). At the end of the first home visit, participants were provided information and instructions for the measures taken in the at‐home period, which included a 5‐day daily monitoring period during which participants provided saliva samples four times per day and completed daily diaries. At the second home visit, anthropometric and health measures were taken. Participants were compensated for their participation. The Wayne State University Institutional Review Board approved all study procedures.

### 2.3 Materials

#### 2.3.1 Lifetime Stressor Severity

Stressor severity across the lifetime was assessed using the Stress and Adversity Inventory for Adults (Adult STRAIN), which is an online system that comprehensively assesses a person's exposure to 55 different major life stressors that are known to impact health (see https://www.strainsetup.com). The system presents one item at a time on a device screen that describes a specific stressor (e.g., car accident, financial difficulties, death of a loved one, discrimination related to race/ethnicity and gender). For each stressor that a participant endorses, a series of follow‐up items are presented, ascertaining the stressor count, type, timing, and severity. Participants rate the severity of each stressor, or how stressful or threatening the stressor was, on a five‐point scale, ranging from “Very slightly or not at all” (1) to “Extremely” (5).

In the HOLD study, a trained research assistant administered the Adult STRAIN as an interview. The STRAIN scores representing lifetime stressor count and severity across the dimensions of exposure timing, primary life domains, and core social‐psychological characteristics were calculated. The present study focuses on indices of stressor severity. Exposure timing is classified as early life stressor severity (the total severity of stressors experienced in the first 18 years of life) or adulthood stress (the total severity of stressors from age 18 through the participant's current age). Lifetime stressor severity encompasses total stress severity experienced from birth through the participant's current age. An additional exposure timing index representing recent life stressor severity was calculated. Consistent with prior studies (e.g., Hostinar et al. 2015), we defined recent life stressors as those occurring within the five years preceding data collection.

Consistent with prior work using the Adult STRAIN (Slavich and Shields 2018), the primary life domain of each stressor was classified as either housing, education, work, treatment/health, marital/partner, reproduction, financial, legal/crime, other relationships, death, life‐threatening situations, or possessions. Similarly, each stressor's primary social‐psychological characteristic was classified as interpersonal loss, physical danger, humiliation, entrapment, or role change/disruption (Slavich and Shields 2018). The Adult STRAIN has excellent concurrent, discriminant, and incremental validity, as well as high test‐retest reliability (*r*_icc_ = 0.936 and 0.953) and strong predictive validity across a variety of psychological, biological, and clinical outcomes (Ahn et al. 2024; Cazassa et al. 2020; Olvera Alvarez et al. 2019; Sturmbauer et al. 2019).

#### 2.3.2 Cortisol

During the 5‐day self‐assessment and saliva collection period, participants provided four saliva samples each day. The samples were provided immediately upon waking, 30 min later to capture the CAR, before dinner, and at bedtime. Cortisol parameters of interest (cortisol at awakening, CAR, and diurnal cortisol slope) were modeled simultaneously using multilevel models. Though individual cortisol parameters such as cortisol slope can be calculated via simple mathematical approaches, multilevel models are recommended to simultaneously estimate multiple parameters (e.g., cortisol at awakening, CAR, and diurnal cortisol slope; Adam and Kumari 2009).

During the self‐assessment period, participants received instructions to store the saliva samples in their home refrigerators, and the research team then retrieved the samples during the second home visit. Following retrieval by study staff, the samples were stored in a −20°C freezer until shipment for analysis. Cortisol concentrations were determined using luminescence immunoassay (IBL, Hamburg, Germany) with intra‐assay and inter‐assay coefficients of variability below 9%. Participants provided 92.6% (*N* = 3761) of the 4060 possible saliva samples. A table summarizing cortisol compliance and cortisol values in each set of analyses can be found in Table 1.

**TABLE 1:** Cortisol compliance and cortisol values across analyses.

	Frequencies (Relative percent of total possible)
Total possible cortisol samples if perfect compliance	4060 (100%)
Cortisol samples provided by participants	3761 (92.6%)
Cortisol samples after noncompliant CAR samples dropped	3423 (84.3%)
Cortisol samples in sensitivity analyses^a^	3216 (79.2%)

^a^ Sensitivity analyses in which samples are dropped when cortisol values are greater than 60 nmol/L or when collected on days participants woke up before 4:00 a.m. or after 11:00 a.m.

#### 2.3.3 Covariates

Person‐level covariates known to influence diurnal cortisol secretion were included in analyses (see Adam and Kumari 2009). These included demographic characteristics, including sex (0 = male, 1 = female; Kirschbaum et al. 1999), age (in years; Ice 2005), marital status (0 = no, 1 = married or cohabitating; Chin et al. 2017), smoking status (0 = never a smoker, 1 = past smoker, 2 = current smoker; Badrick et al. 2007), individual SES (Cohen et al. 2006), and self‐reported chronic health conditions (Kudielka and Kirschbaum 2003).^1^ SES was computed by averaging participants’ *z*‐scored highest level of educational attainment (on a scale from 1 = no school or some grade school to 12 = Ph.D. or other advanced degree) and household income (from 1 = < $5000 to 13 = ≥ $150,000). Despite there being some conceptual overlap between SES and financial stressor count and severity as measured by the STRAIN, SES was only modestly correlated with financial stressor severity (*r* = −0.15, *p* = 0.036) and was not significantly correlated with financial stressor count (*r* = −0.053, *p* = 0.454), indicating a lack of potential multicollinearity between the variables.

The presence of self‐reported chronic health conditions was assessed by asking participants whether they experienced any chronic conditions out of a list of 16 conditions (0 = no conditions, 1 = one chronic health condition, 2 = two or more chronic health conditions) over the last 12 months. Three daily‐level covariates related to cortisol collection (i.e., wake‐up time, medication use, day of the week [0 = weekday, 1 = weekend]) were included. Daily medication use was self‐reported each day for 5 days as part of the daily diary protocol. For daily medication use, participants were asked to list the name of their medication, the dose, the number of times taken that day, and what time each medication was taken. Responses on medication use for each day were coded as 0 = no (no medication taken that day), and 1 = yes (at least one medication taken that day).

### 2.4 Analytic Strategy

Analyses were performed in Mplus 7.0 (Muthén and Muthén 2012). Consistent with Zilioli et al. (2023), hypotheses were tested using three‐level multilevel models (MLMs). Time since waking, quadratic time since waking, and CAR (1 = 30‐min post‐waking sample; 0 = all others) were included at Level 1 (cortisol‐level). Sampling times were recorded using MEMs 6 TrackCap Monitors (Aardex Ltd., Switzerland) and subsequently used to calculate time since waking. When this data was unavailable, self‐reported sampling times were used to calculate these values. When neither were available, we used MEMs person‐means for each sampling time point, and we used MEMs grand‐means for each sampling time point if MEMs person‐means were unavailable. If CAR samples exceeded the requested 30‐min sampling time by 10 min or more, the CAR samples were dropped from analyses (33.2% of available CAR samples; e.g., Adam et al. 2015; Kudielka et al. 2003). Once noncompliant CAR values were dropped, 3423 cortisol values remained in the analytic sample. Quadratic time since waking was modeled as a fixed effect; cortisol at awakening (intercept), cortisol slope, and CAR were modeled as random effects. Day‐level variables (wake‐up time, day of the week [0 = weekdays, 1 = weekends], and medication use [0 = no, 1 = yes]) were included as predictors at Level 2. Person‐level variables of interest (stressor severity across lifetime, early life, recent life, or the interaction between early life and recent life stressors) and person‐level control variables were included at Level 3.

MLMs were run separately to test the biological embedding, cumulative, and sensitization hypotheses. Each model was run first with only Level 2 covariates and then with Level 2 and Level 3 covariates. Consistent with work by Young et al. (2019), the model testing the cumulative stress model included lifetime stressor severity as a predictor. The model testing the biological embedding hypothesis included early life stressor severity as a predictor. The model testing the sensitization model included early life stressor severity, recent life stressor severity, and their interaction as predictors. A hierarchical approach was taken to sensitization analyses. In Model A, only the main effects of early life and recent life stressor severity were included. In Model B, the main effects and the interaction were included. All Level‐3 continuous variables were grand‐mean centered prior to analyses. To correct for positive skewness, cortisol values were natural log‐transformed in all models. We added a constant of 1 prior to transformation so that transformed values remained positive.

Following prior studies (Karlamangla et al. 2019; Zilioli et al. 2023), sensitivity analyses were conducted. In sensitivity analyses, the primary models above were rerun after dropping cortisol values > 60 nmol/L to reduce the potential influence of high values (e.g., Karlamangla et al. 2019) and dropping values collected on days participants woke prior to 4:00 a.m. or after 11:00 a.m., as extreme wake‐up times can influence diurnal cortisol secretion throughout the day (Edwards et al. 2001; Karlamangla et al. 2019). See Table 1 for compliance rates and cortisol values across analyses.

The primary analyses focus on the severity of early life, cumulative, and current stressor events rather than the count of stressor events. Though this approach is consistent with methodology from Young et al. (2019), theoretical models, such as the cumulative stress model, frequently focus on stressor count rather than severity (Evans et al. 2013; Slopen et al. 2018). Therefore, in sensitivity analyses, primary analyses were also rerun with stressor event count rather than severity for lifetime stressors, early life stressors, and recent life stressors included at Level 3 to test whether the pattern of effects differed. We also conducted exploratory analyses examining bivariate correlations between diurnal cortisol parameters and lifetime stressor severity in the specific domains (e.g., housing, education, etc.) and social‐psychological characteristics (e.g., interpersonal loss, physical danger, etc.) described above.

Missing data at Level 1 (15.7%) were handled using full‐information maximum likelihood (FIML). Missing data at Level 2 (8.47% missing data among Level 2 variables, affecting wake‐up time) were replaced with the participant's mean value of the corresponding data completed on other days. When this information was unavailable, missing data were replaced with the mean values of the total sample (e.g., Zilioli et al. 2023; Zilioli and Jiang 2021). When missing data was present at Level 3 (a total of 1.52% missing data among Level 3 variables), missing values for continuous variables were imputed using the expectation‐maximization algorithm, and missing values for categorical variables were imputed using mode imputation.

## 3 Results

### 3.1 Descriptives

Sample descriptive statistics and bivariate correlations between person‐level variables are displayed in Table 2. The average score was 72.61 (SD = 35.04) for lifetime stressor severity, 10.13 (SD = 9.50) for early life stressor severity, and 8.06 (SD = 8.62) for recent life stressor severity. Total lifetime stressor severity was moderately correlated with early (*r* = 0.55, *p* < 0.001) and recent life stressor severity (*r* = 0.50, *p* < 0.001). Early and recent life stressor severity were weakly correlated (*r* = 0.18, *p* = 0.016).

**TABLE 2:** Mean, standard deviations, and correlations of study variables.

Variables	1	2	3	4	5	6	7	8	9
1. Lifetime stressor severity	—								
2. Early life stressor severity	0.55***	—							
3. Recent stressor severity	0.49***	0.17*	—						
4. Socioeconomic status	−0.11	0.02	−00.12	—					
5. Age	−0.21**	−0.16*	−0.38***	0.30***	—				
6. Sex	−0.02	−0.12	−0.11	0.15*	0.33***	—			
7. Marital status	0.04	0.06	0.07	0.11	−0.08	0.18*	—		
8. Smoking status	0.07	0.06	0.12	−0.26***	−0.30***	−0.37***	0.12	—	
9. Number of chronic conditions	0.25*	0.02	0.10	−0.02	0.18**	0.26***	−0.03	−0.11	—
*M*	72.52	10.12	8.02	0.00	67.53	0.72	0.19	0.73	1.25
SD	34.34	9.30	8.46	0.89	8.38	0.45	0.39	0.82	1.26

Abbreviation: CAR = cortisol awakening response.

* *p* < 0.05; **p < 0.01; ***p < 0.001.

### 3.2 Primary Analyses

#### 3.2.1 Cumulative Model

Results from the unadjusted cumulative model showed that lifetime stressor severity was significantly associated with blunted CAR (*b* = −0.002, SE = 0.001, *p* = 0.042) but not with diurnal cortisol slope (*b* = 0.000, SE = 0.000, *p* = 0.177) or cortisol at awakening (*b* = 0.000, SE = 0.002, *p* = 0.806). However, after controlling for covariates at Level 3 (see Table 3), associations between lifetime stressor severity and CAR were no longer significant (*p* = 0.063).

**TABLE 3:** Results of multilevel models testing effects of stressor severity on diurnal cortisol parameters.

	Cumulative model		Biological embedding model		Sensitization model A		Sensitization model B	
Fixed effects	Coefficient (SE)	*P*	Coefficient (SE)	*P*	Coefficient (SE)	*p*	Coefficient (SE)	*p*
Cortisol at awakening, π0								
Average cortisol at awakening, β00, γ000	1.656 (0.212)	< 0.001	1.654 (0.212)	< 0.001	1.662 (0.213)	< 0.001	1.605 (0.218)	< 0.001
Female, γ001	0.159 (0.121)	0.189	0.164 (0.123)	0.183	0.164 (0.124)	0.183	0.190 (0.214)	0.125
Marital status, γ002	−0.341 (0.094)	< 0.001	−0.344 (0.094)	< 0.001	−0.347 (0.094)	< 0.001	−0.339 (0.095)	< 0.001
Age, γ003	−0.010 (0.007)	0.171	−0.009 (0.007)	0.200	−0.007 (0.007)	0.335	−0.007 (0.007)	0.339
Smoking, γ004	−0.008 (0.074)	0.911	−0.008 (0.073)	0.911	−0.009 (0.073)	0.907	−0.003 (0.074)	0.970
Chronic health conditions, γ005	−0.006 (0.058)	0.923	−0.013 (0.059)	0.831	−0.016 (0.060)	0.786	−0.012 (0.060)	0.847
Socioeconomic status, γ006	0.080 (0.052)	0.121	0.079 (0.051)	0.123	0.080 (0.052)	0.124	0.071 (0.053)	0.181
Lifetime stressor severity γ007	0.000 (0.002)	0.779	**—**	**—**	**—**	**—**	**—**	**—**
Early life stressor severity γ007	**—**	**—**	0.003 (0.007)	0.665	0.002 (0.007)	0.730	−0.003 (0.008)	0.682
Recent stressor severity γ008	**—**	**—**	**—**	**—**	0.005 (0.008)	0.552	−0.004 (0.012)	0.760
Early x recent stressor severity γ009	**—**	**—**	**—**	**—**	**—**	**—**	0.001 (0.001)	0.243
Day of the week, β01, γ010	−0.013 (0.043)	0.753	−0.014 (0.043)	0.749	−0.014 (0.043)	0.749	−0.014 (0.043)	0.750
Wake‐up time, β02, γ020	−0.004 (0.017)	0.819	−0.004 (0.018)	0.803	−0.005 (0.018)	0.782	−0.005 (0.017)	0.787
Medication use, β03, γ030	−0.099 (0.069)	0.150	−0.099 (0.070)	0.155	−0.106 (0.068)	0.118	−0.103 (0.068)	0.129
CAR, π1								
Average CAR, β10, γ100	0.165 (0.146)	0.260	0.187 (0.148)	0.205	0.180 (0.145)	0.216	0.172 (0.147)	0.242
Female, γ101	0.041 (0.073)	0.573	0.039 (0.074)	0.599	0.040 (0.073)	0.587	0.043 (0.073)	0.559
Marital status, γ102	0.074 (0.073)	0.313	0.071 (0.073)	0.328	0.071 (0.071)	0.318	0.071 (0.071)	0.314
Age, γ103	0.000 (0.005)	0.999	0.001 (0.005)	0.785	−0.001 (0.005)	0.910	−0.001 (0.005)	0.898
Smoking, γ104	0.033 (0.043)	0.441	0.032 (0.044)	0.462	0.032 (0.043)	0.460	0.033 (0.044)	0.449
Chronic health conditions, γ105	−0.048 (0.040)	0.228	−0.059 (0.040)	0.139	−0.056 (0.040)	0.155	−0.055 (0.040)	0.163
Socioeconomic status, γ106	−0.004 (0.037)	0.918	0.005 (0.037)	0.896	0.004 (0.037)	0.922	0.002 (0.038)	0.957
Lifetime stressor severity γ107	−0.002 (0.001)	0.063	**—**	**—**	**—**	**—**	**—**	**—**
Early life stressor severity γ107	**—**	**—**	−0.003 (0.003)	0.409	−0.002 (0.003)	0.475	−0.003 (0.005)	0.454
Recent stressor severity γ108	**—**	**—**	**—**	**—**	−0.004 (0.004)	0.248	−0.006 (0.006)	0.316
Early x recent stressor severity γ109	**—**	**—**	**—**	**—**	**—**	**—**	0.000 (0.000)	0.748
Day of the week, β11, γ110	−0.024 (0.044)	0.587	−0.026 (0.044)	0.557	−0.026 (0.044)	0.560	−0.025 (0.044)	0.567
Wake‐up time, β12, γ120	−0.027 (0.018)	0.139	−0.027 (0.018)	0.138	−0.026 (0.018)	0.154	−0.026 (0.018)	0.156
Medication use, β13, γ130	0.004 (0.055)	0.943	−0.006 (0.057)	0.910	−0.004 (0.058)	0.948	−0.006 (0.057)	0.923
Time since waking, π2								
Average linear slope, β20, γ200	−0.087 (0.013)	< 0.001	−0.088 (0.013)	< 0.001	−0.088 (0.013)	< 0.001	−0.089 (0.013)	< 0.001
Female, γ201	−0.010 (0.006)	0.075	−0.010 (0.006)	0.093	−0.010 (0.006)	0.086	−0.009 (0.006)	0.101
Marital status, γ202	0.006 (0.005)	0.285	0.006 (0.005)	0.282	0.006 (0.005)	0.269	0.006 (0.005)	0.261
Age, γ203	0.000 (0.000)	0.999	0.000 (0.000)	0.992	0.000 (0.000)	0.784	0.000 (0.000)	0.788
Smoking, γ204	0.006 (0.003)	0.062	0.006 (0.003)	0.057	0.006 (0.003)	0.056	0.006 (0.003)	0.050
Chronic health conditions, γ205	−0.003 (0.003)	0.264	−0.003 (0.003)	0.320	−0.003 (0.003)	0.346	−0.003 (0.003)	0.360
Socioeconomic status, γ206	−0.006 (0.003)	0.045	−0.006 (0.003)	0.025	−0.006 (0.003)	0.024	−0.006 (0.003)	0.023
Lifetime stressor severity γ207	0.000 (0.000)	0.223	**—**	**—**	**—**	**—**	**—**	**—**
Early life stressor severity γ207	**—**	**—**	0.000 (0.000)	0.118	0.000 (0.000)	0.096	0.000 (0.000)	0.342
Recent stressor severity γ208	**—**	**—**	**—**	**—**	0.000 (0.000)	0.407	0.000 (0.000)	0.353
Early x recent stressor severity γ209	**—**	**—**	**—**	**—**	**—**	**—**	0.000 (0.000)	0.684
Day of the week, β21, γ210	−0.001 (0.004)	0.763	−0.001 (0.004)	0.769	−0.001 (0.004)	0.770	−0.001 (0.004)	0.769
Wake‐up time, β22, γ220	0.000 (0.001)	0.782	0.000 (0.001)	0.739	0.000 (0.001)	0.718	0.000 (0.001)	0.714
Medication use, β23, γ230	0.003 (0.005)	0.591	0.003 (0.005)	0.491	0.004 (0.005)	0.428	0.004 (0.005)	0.423
Time since waking2, π3								
Average curvature, γ300	0.004 (0.000)	< 0.001	0.004 (0.000)	< 0.001	0.004 (0.000)	< 0.001	0.004 (0.000)	< 0.001

Abbreviation: CAR = cortisol awakening response.

#### 3.2.2 Biological Embedding Model

Results from the biological embedding model showed that early life stressor severity was not significantly associated with diurnal cortisol slope (*b* = 0.000, SE = 0.000, *p* = 0.096), CAR (*b* = −0.003, SE = 0.003, *p* = 0.327), or cortisol at awakening (*b* = 0.003, SE = 0.007, *p* = 0.691). Controlling for covariates at Level 3 did not alter inferences of these results (see Table 3).

#### 3.2.3 Sensitization Model

Results from Sensitization Model A showed no main effects of early and recent life stressor severity on diurnal cortisol slope (Early: *b* = 0.000, SE = 0.000, *p* = 0.093; Recent: *b* = 0.000, SE = 0.000, *p* = 0.960), CAR (Early: *b* = −0.003, SE = 0.003, *p* = 0.412; Recent: *b* = −0.004, SE = 0.003, *p* = 0.255), or cortisol at awakening (Early: *b* = 0.002, SE = 0.007, *p* = 0.782; Recent: *b* = 0.005, SE = 0.008, *p* = 0.532). Similarly, Sensitization Model B showed that the interaction between early and recent life stressor severity was not significant for any of the cortisol parameters (diurnal cortisol slope [*b* = 0.000, SE = 0.000, *p* = 0.734], CAR [*b* = 0.000, SE = 0.000, *p* = 0.693], or cortisol at awakening [*b* = 0.001, SE = 0.001, *p* = 0.219]). Results did not differ when controlling for covariates at Level 3 (see Table 3).

#### 3.2.4 Bivariate Associations

The lifetime severity of stressors involving education was significantly associated with blunted diurnal cortisol slope (*b* = 0.007, SE = 0.003, *p* = 0.035) and lower cortisol at awakening (*b* = −0.159, *SE* = 0.075, *p* = 0.034) but was unrelated to CAR (*b* = −0.002, SE = 0.049, *p* = 0.965). The severity of stressors involving non‐marital relationships was related to blunted CAR (*b* = −0.472, SE = 0.233, *p* = 0.043) but not diurnal cortisol slope (*b* = 0.019, SE = 0.020, *p* = 0.341) or cortisol at awakening (*b* = −0.105, SE = 0.420, *p* = 0.802). The severity of stressors involving housing, work, medical treatments/health, marital relationships, reproduction, finances, legal systems/crime, death, life‐threatening situations, and possessions were not significantly related to any of the three cortisol parameters (*p*s > 0.052). The severities of stressors characterized by danger, interpersonal loss, humiliation, entrapment, and role changes were each unrelated to all three cortisol parameters (*p*s > 0.051).

### 3.3 Sensitivity Analyses

#### 3.3.1 Theoretical Models

In the sensitivity analyses for the cumulative model, the effect of lifetime stressor severity on diurnal cortisol parameters remained nonsignificant when cortisol values greater than 60 nmol/L or collected on days participants woke up prior to 4:00 a.m. or after 11:00 a.m. were dropped. For the biological embedding model, the effects of early life stressor severity on cortisol parameters remained nonsignificant. Regarding the sensitization model, no significant main effects or interactions between early and recent life stressors emerged for any cortisol parameters (*p*s > 0.052).

**FIGURE 1:**
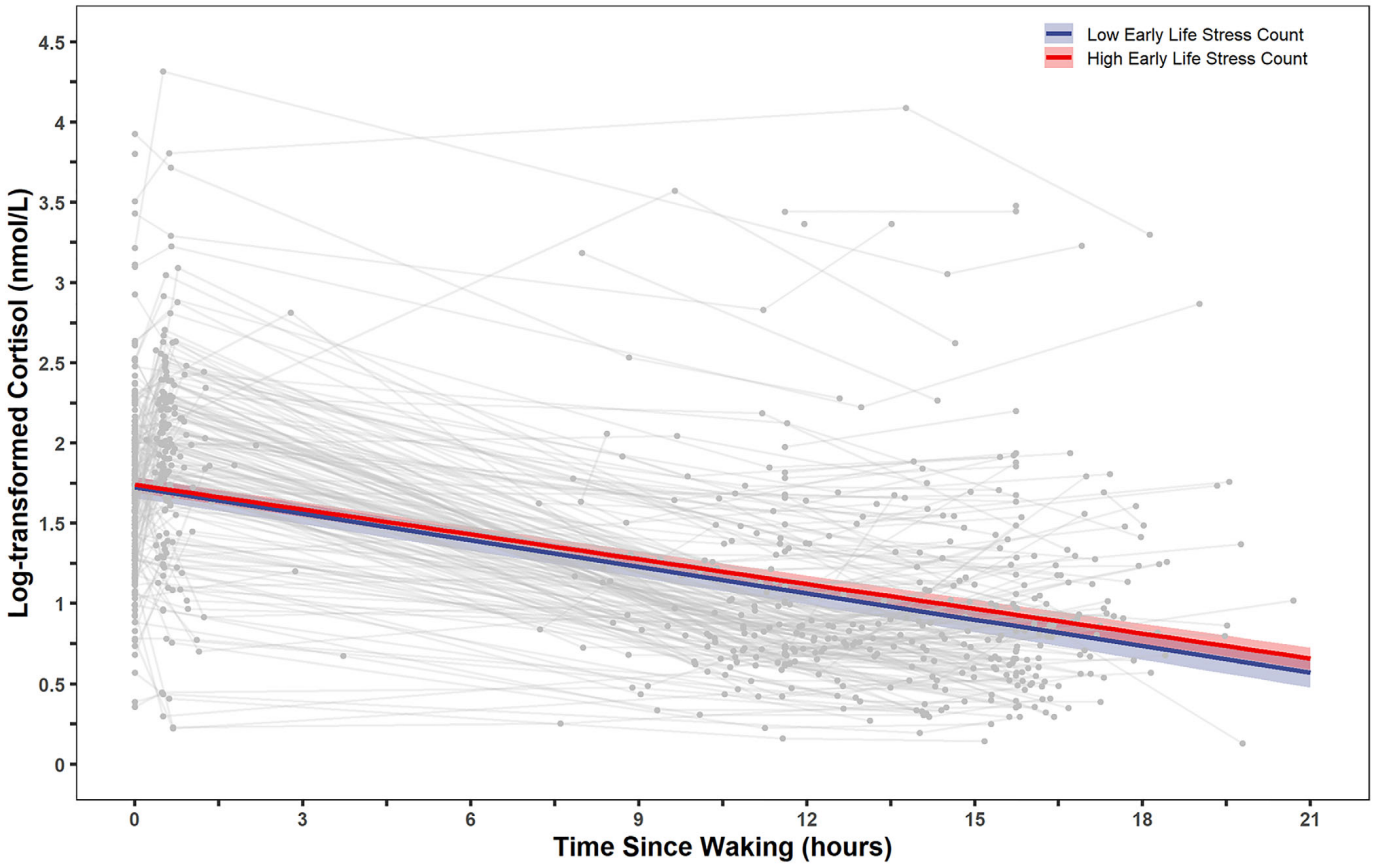
Early life stressor count and diurnal cortisol (log‐transformed), as a function of time since waking. The line depicting high early life stress count (red) refers to values 1 standard deviation above the sample mean, and the line depicting low early life stress (blue) refers to values 1 standard deviation below the sample mean. The grey lines and points represent the raw data.

Examination of the count, rather than severity, of stressors did not meaningfully alter the results. When cortisol values greater than 60 nmol/L were dropped or collected on days participants woke up before 4:00 a.m. or after 11:00 a.m. from the analyses with stressor count, results for the cumulative model were nonsignificant. However, when the biological embedding model was run with early life stressor count rather than severity, early life stressor count emerged as a significant predictor of blunted diurnal cortisol slope (*b* = 0.001, SE = 0.001, *p* = 0.032; see Figure 1). In Sensitization Model A, the main effect of early life stressor count was significantly once again associated with a blunted diurnal cortisol slope (*b* = 0.002, SE = 0.001, *p* = 0.029). In Sensitization Model B, the interaction remained nonsignificant (*p*s > 0.055).

#### 3.3.2 Bivariate Associations

When cortisol values greater than 60 nmol/L or collected on days participants woke up prior to 4:00 a.m. or after 11:00 a.m. were dropped, stressor severity related to educational domains remained significantly associated with blunted diurnal cortisol slopes (*b* = 0.008, SE = 0.003, *p* = 0.006) and blunted cortisol at awakening (*b* = −0.130, SE = 0.061, *p* = 0.033). The severity of stressors involving non‐marital relationships was no longer associated with blunted CAR (*b* = −0.444, SE = 0.230, *p* = 0.054). Stressor severity in the domain of life‐threatening situations became significantly associated with a blunted diurnal cortisol slope (*b* = 0.034, SE = 0.015, *p* = 0.022). Patterns of results for stressors involving work, housing, medical treatments/health, marital relationships, reproduction, finances, legal systems/crime, death, and possessions were unchanged in sensitivity analyses, as were the patterns of results for stressor severity characterized by interpersonal loss, danger, humiliation, entrapment, and role change/disruptions.

## 4 Discussion

We tested three competing models of the association between stressor severity and diurnal cortisol parameters in a sample of older African American adults: the cumulative model (Karatsoreos and McEwen 2013; McEwen 1998, 2008), the biological embedding model (Doom and Gunnar 2013; Lupien et al. 2009; Shonkoff et al. 2009), and the sensitization model (Daskalakis et al. 2013). In our primary analyses, the data were not consistent with the predictions of any of these models. Although lifetime stressor severity was associated with blunted CAR in unadjusted models, this association was no longer significant once models were adjusted for covariates, indicating that these analyses do not robustly support the cumulative model. This pattern of results was unchanged when lifetime stress count, rather than severity, was examined. However, when focusing on diurnal cortisol slope—the cortisol parameter most consistently associated with stress and health outcomes (Adam et al. 2017)—our sensitivity analyses revealed that early life stressor count was significantly associated with blunted diurnal cortisol slope when cortisol values greater than 60 nmol/L or collected on days participants woke up prior to 4:00 a.m. or after 11:00 a.m. were dropped. Further, exploratory analyses provided preliminary support for a stressor characteristics approach (Lam et al. 2019; Slavich et al. 2009, Slavich et al. 2010; Slavich and Shields 2018), such that some stressor characteristics were more salient predictors of some cortisol parameters.

These findings differ from prior studies that tested competing models of stressors across developmental periods on stress‐related physiology. Young et al. (2019) found support for the sensitization model. Specifically, they found that the confluence of high early‐life stressor exposure and high current stressor exposure predicted significantly flatter diurnal cortisol slopes. However, it is important to note that the study differed meaningfully from the present investigation. Although Young and colleagues examined longitudinal stressor associations with cortisol, their participants were all 37 years old, thus significantly younger than the participants in our study. Further, the majority of the participants in the study were White, whereas the participants in the present study were African American.

Prior studies have found that, compared to White Americans, African Americans are more likely to be exposed to a variety of chronic stressors (Brown et al. 2020), have flatter diurnal cortisol slopes, and have cortisol levels that are less robustly associated with stressor exposures (Skinner et al. 2011). For example, Skinner and colleagues examined the associations between stressor exposure and diurnal cortisol slope in African American and White participants. They found that exposure to stressors was a significant predictor of blunted diurnal cortisol slopes in White participants, but not African American participants. Similarly, Cohen et al. (2006) and DeSantis et al. (2007) identified significant racial/ethnic differences in diurnal cortisol slopes. These differences were not mediated by psychosocial factors, including experiences of chronic strains or measures of chronic and episodic stressors, respectively. It is important to note, however, that one study found associations between lifetime stressors and diurnal slopes in African American adults, such that high levels of lifetime stressors were associated with flatter diurnal cortisol slopes in African American pregnant women (Suglia et al. 2010). Although these studies did not explicitly evaluate any of the three competing hypotheses examined here, our study is broadly consistent with prior work, which mostly did not find associations between stressor severity and diurnal cortisol in African American adults. It is also possible that existing stress exposure and severity measures do not adequately capture the stressors most relevant to diurnal cortisol regulation in African Americans (e.g., Zilioli et al. 2023).

In contrast to the studies that did not find associations between life stressors and diurnal cortisol in African Americans, some preliminary support for the biological embedding model emerged from our sensitivity analyses. To remain as consistent as possible with work by Young et al. (2019), our primary analyses focused on stressor severity. However, many theoretical models (e.g., cumulative model) frequently emphasize lifetime stressor count rather than severity (Evans et al. 2013; Slopen et al. 2018). Therefore, in our sensitivity analyses, we examined stressor counts as primary predictors in each model. Results of our sensitivity analyses suggested that early life stressor count, but not severity, may be associated with blunted diurnal cortisol slopes. Prior research has suggested that, although African American adults report significantly more stressor exposure than White Americans, African Americans tend to appraise stressors as less upsetting than White Americans (Brown et al. 2020). Brown and colleagues argue that this distinction in stress appraisal across races may help elucidate pathways contributing to racial health disparities. The authors present several hypotheses for these racial differences in stress appraisal, including elevated engagement in active reframing among minoritized groups, which might downplay perceptions of stress without necessarily curtailing its physiological effects. As such, measurements of stressor appraisals may differentially capture the impact of stressors across racial groups, which may, therefore, partially explain why stressor count might emerge as a better predictor of diurnal cortisol slope than stressor severity (Lebois et al. 2016). Our results should be interpreted cautiously as they emerged from sensitivity analyses rather than primary analyses. For example, these results may indicate that the association between early life stressor count and diurnal cortisol slope might be sensitive to extreme values due to the modest sample size of this study.

Although the association between early life stressor count and diurnal cortisol slopes requires replication and should be interpreted with caution, it is consistent with previous findings in multiracial samples noting significant associations between childhood adversity and flattened diurnal cortisol slopes in adulthood (Karlamangla et al. 2019; Weissbecker et al. 2006). Additionally, one study focusing on African American women found significant associations between higher childhood stressor exposure and higher levels of mid‐day plasma cortisol (Gillespie et al. 2017). However, it is important to note that a single measurement of plasma cortisol does not allow for the characterization of diurnal cortisol parameters, such as cortisol at awakening, CAR, or slope. As such, there remains a dearth of research examining associations between childhood stressor exposure and diurnal cortisol patterns in African American adults.

We also found preliminary support for the associations between diurnal cortisol parameters and the characteristics and domains of stressors experienced. Stressor severity related to the domain of education was significantly associated with blunted diurnal cortisol slope in exploratory and sensitivity analyses. Associations between education‐related stressors and cortisol have been previously noted in the literature (Bai et al. 2017; Malanchini et al. 2021). A study of children and adolescents found that school‐related achievement was significantly associated with a latent variable indicated by diurnal cortisol slope, hair cortisol, and cortisol reactivity and recovery to a brief stressor (Malanchini et al. 2021). Another study of cortisol secretion in children found that experiences of academic problems were related to higher cortisol at awakening but unrelated to diurnal slopes (Bai et al. 2017). In contrast, college‐related stress was associated with a lower CAR in Latino/a college students (Sasser et al. 2023). However, the effects of education‐related stressors in adults, and particularly African American adults, beyond the college years remain understudied.

In sensitivity analyses, stressor severity in the domain of life‐threatening situations was also associated with a blunted diurnal cortisol slope. Exposure to life‐threatening and traumatic events in children and adults has been previously associated with alterations in cortisol secretion (Kinney et al. 2023; Klaassens et al. 2010; Kuras et al. 2017; Schreier et al. 2016). In analyses stratified by participant race, Schreier and colleagues found associations between traumatic life events and elevated hair cortisol concentrations among only African American participants. Consistent with these studies, our work suggests that both education‐related stressor severity and stressor severity associated with life‐threatening situations may be related to a blunted diurnal cortisol slope in older African American adults. However, it is important to note that these exploratory findings require replication. Overall, the current findings add to evidence that specific domains of stress, such as experiences of discrimination (see also Zilioli et al. 2023), appear significantly associated with diurnal cortisol in African American adults.

### 4.1 Strengths and Limitations

Several strengths of this study should be noted. First, we systematically assessed a variety of major life stressors occurring across the entire life course, which is critical for testing theories of stress and health (Shields and Slavich 2017; Slavich 2016, 2019). Second, analyses were preregistered and focused on comparatively testing several leading theories of stress and health. Finally, we examined the effects of stressor severity on cortisol, a biologically plausible mechanism mediating the effects of stress on health.

In addition, there are several limitations. First, compliance for CAR samples was monitored by comparing sample times as recorded by MEMs 6 TrackCap Monitors. While this is a commonly used approach (Young et al. 2019; Zilioli et al. 2023), it is possible that the first sample was not taken immediately upon waking, which may have slightly influenced our calculations of time since waking and the 30‐min windows for CAR compliance. Furthermore, the majority of the sample was female (72.4%), which may limit the generalizability of the findings to older African American male adults or other demographic groups. The higher proportion of female participants is somewhat unsurprising as women tend to participate in research more frequently than men (Glass et al. 2015; Wild et al. 2001). It is important to note, however, that some meta‐analytic work suggests that sex does not moderate associations between stressors like discrimination and cortisol output (Korous et al. 2017). Regardless, efforts to recruit older male African American adults are needed in future research to better understand the link between stressor severity, stressor frequency, and cortisol dynamics in this population and fully examine the role of sex in these associations.

Finally, diurnal cortisol and stressor severity across the lifespan were measured concurrently, and stressor severity at previous developmental periods (e.g., early life stressor severity) was assessed retrospectively. Prospective, longitudinal studies of stressor severity, stressor count, and cortisol across the lifespan are encouraged to elucidate the temporal dynamics of their associations. (Doom and Gunnar 2013). A more fine‐grained examination of early life stressor severity, count, and cortisol parameters in older African American adults is needed to replicate the current results and identify if and when early life stressors most strongly predict cortisol secretion in later life. Lastly, longitudinal studies are needed to prospectively assess the contributions of the characteristics and frequencies of stressors to diurnal cortisol slopes in older African American adults.

### 4.2 Conclusion

Notwithstanding these limitations, the present data are the first to test three conceptual models of stressor severity across the lifetime and diurnal cortisol parameters in a sample of older African American adults. Specifically, in a sample of African Americans over the age of 50 living in Detroit, we found no support for any of the three models of stressor *severity* across the lifetime and diurnal cortisol parameters. Instead, we found preliminary support for the association between early life stressor *count* and diurnal cortisol slope and support for a stressor characteristics approach. Future research is needed to assess stressor exposure and severity prospectively and longitudinally across the lifespan and its relation to diurnal cortisol dynamics in African American adults.

## Declaration of AI‐Assisted Technologies in the Writing Process

During the preparation of this work, ChatGPT (https://chat.openai.com/chat) was used for editing purposes. After using this tool/service, the authors reviewed and edited the content as needed and take full responsibility for the content of the publication.

## Conflicts of Interest

The authors declare no conflicts of interest.

## Data Availability

The data that support the findings of this study are available from the corresponding author upon reasonable request.

## Endnotes

1 Coding of three covariates differed from the pre‐registered analyses. Rather than being coded dichotomously, smoking and chronic health conditions were categorized into one of three categories to better capture variability within the sample. Rather than being coded as married versus all others, marital status was coded as married/cohabitating versus all others to better capture romantic companionship, rather than legal marriage alone.
